# The Association of New-Onset Atrial Fibrillation and Risk of Cancer: A Systematic Review and Meta-Analysis

**DOI:** 10.1155/2020/2372067

**Published:** 2020-09-27

**Authors:** Mengxia Zhang, Lin-ling Li, Qian-qian Zhao, Xiao-dong Peng, Kui Wu, Xin Li, Yan-Fei Ruan, Rong Bai, Nian Liu, Chang Sheng Ma

**Affiliations:** Department of Cardiology, Beijing Anzhen Hospital, Capital Medical University, National Clinical Research Center for Cardiovascular Diseases, No. 2 Anzhen Road, Chaoyang District, Beijing 100029, China

## Abstract

**Background:**

There are distinct results for the relationship between new-onset atrial fibrillation (NOAF) and subsequent incident cancer. To date, no systematic analysis has been conducted on this issue. This study aims to explore the relationship between NOAF and the risk of developing cancer through a meta-analysis with a large sample size.

**Methods:**

Electronic databases, such as PubMed and EMBASE, were searched for published relevant studies on NOAF patients diagnosed with cancer after and during follow-ups, including reported records of baseline information and the statistical result of morbidity. Two investigators independently reviewed the articles and extracted the data using uniform standards and definitions. The meta-analysis was conducted using the Cochrane Program Review Manager.

**Results:**

This meta-analysis consisted of five cohort studies and one case-control study, which comprised 533,514 participants. The pooled relative risk (RR) for incident cancer was 1.24 (95% CI: 1.10–1.39, *P*=0.0003). The temporal trend analysis demonstrated that an increased risk of cancer was observed during the initial 90 days (RR: 3.44, 95% CI: 2.29–5.57, *P* < 0.00001), but not after that. Lung cancer (RR: 1.51, 95% CI: 1.47–1.55, *P* < 0.00001) was associated with NOAF, but not colorectal cancer and breast cancer.

**Conclusion:**

This meta-analysis provides evidence that NOAF is associated with increased risk of cancer. The risk of incident cancer particularly increases within 90 days after NOAF diagnosis, but not after that.

## 1. Introduction

It has been well recognized that the new diagnosis of cancer would promote the subsequent development of new-onset atrial fibrillation (NOAF) [1]. The underlying mechanisms may be correlated with co-risk factors underlying the two independent disease entities and the medical interventions for cancer, such as chemotherapy and radiotherapy, which cause cardiotoxicity and predispose these patients to atrial fibrillation (AF).

Recently, several studies have demonstrated that NOAF may increase the risk of incident cancer, thereby shedding light on the mutual interactions between AF and cancer [2–4]. However, not all studies are in agreement with this association. AF is the most common type of sustained tachyarrhythmia encountered in clinical practice. Comorbid cancers in patients with NOAF significantly result in the complexity of clinical management and contribute to poor clinical outcomes [5]. Some AF trials have demonstrated that malignancy is the leading cause of death among noncardiovascular deaths [6, 7]. The exploration of the link between NOAF and subsequent cancer is critical for the establishment of risk stratification and early intervention for patients with NOAF. The objective of the present meta-analysis was to determine whether NOAF increases the risk of development of cancer.

## 2. Methods

### 2.1. Search Strategy

**Figure 1:**
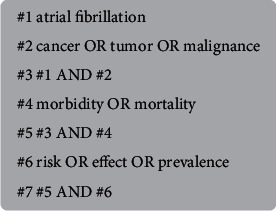
An example of the PubMed retrieval strategy.

According to the recommendations of the Meta-analysis of Observational Studies in Epidemiology Group [8], relevant English language articles were searched from electronic databases (PubMed, EMBASE, and Cochrane Library) updated to April 2020. All related MeSH headings and text search strategies were used with the following keywords: atrial fibrillation (AF), cancer (tumor and malignancy), morbidity (mortality), and relative risk (RR) or odds ratio (OR) or hazard ratio (HR). One particular instance is presented in Figure 1. The reference list of the published articles was manually checked to identify any additional studies.

### 2.2. Selection Criteria

The present study aimed to determine whether NOAF patients have a higher risk of developing cancer. Studies related to patients who have AF or cancer history were excluded. Studies that enrolled subjects based on patients with a specific disease condition or with unadjusted risks for associated events were further abnegated. If multiple studies were derived from the same cohort and covered by similar events, only the most complete studies and latest published information were incorporated for the present primary analysis. All ideal evidence should meet the following criteria: (1) observational studies with appropriate follow-up; (2) studies that shared the standard definition of AF and cancer patterns; (3) the included subjects were healthy, and the baseline raw data were generally comprehensive; (4) necessary information, such as the incident cancer reports of adjusted results and risk ratio (RR), odds ratio (OR), and hazard ratio (HR), was clearly expressed. Studies were excluded based on the following: (1) the articles were case reports, reviews, or basic research studies; (2) the data of the study were incomplete or duplicated.

### 2.3. Quality Assessment

Screening, data extraction, and critical appraisal were independently undertaken by two reviewers. In order to rule out irrelevant or repeating articles, the investigators perused the content of the remaining studies and assessed the quality of each report. Any possible divergence or indetermination was settled by discussion or arbitration with a third referee. For each eligible study, the Newcastle–Ottawa Quality Assessment Scale (NOS) was used to evaluate the quality and obtain the final scores. With a total rating of nine stars, a study that scores higher than or equal to seven stars was defined as high-quality research. Otherwise, the study was defined as low-quality research.

### 2.4. Data Extraction

The raw data were extracted, which included the following: (1) the necessary information of the qualified literature, such as the first author's name, publication time, region difference, and type of research; (2) the key elements to evaluate the risk of inclusion bias, such as disease definition, subgroup criteria, and the final score of NOS; (3) the medical details of subjects, with or without AF, and before or after the occurrence of cancer; (4) the significant outcome indicators at the end of the study. Furthermore, the RRs, HRs, ORs, and 95% confidence intervals (CIs) that were preferentially multivariate adjusted, rather than age/gender adjusted, from separate articles were extracted to assess the relevance between AF and cancer.

### 2.5. Data Synthesis and Statistical Analysis

The data used for the present meta-analysis were based on the adjusted outcome from every included study and were logarithmically transformed. In addition, the corresponding standard errors (SE) were calculated and combined with the log relative risk using the inverse variance approach. The original HR/OR value in articles from the multivariate Cox proportional hazards model was regarded as the approximate RR. The *I*^2^-test and *Q* statistics were used to quantitatively determine the heterogeneity. If there was no statistical heterogeneity among the results (i.e., *P*_Q statistic_ > 0.1 and *I*^2^ ≤ 50%), the fixed-effect model can be adopted for the meta-analysis. Otherwise, the random-effect model was applied. This was due to the clinical and methodological differences between studies. Subgroup analyses for the main indicators, such as gender and the subtype of cancer, as well as the time interval between NOAF diagnosis and cancer, were conducted to search for heterogeneity sources. When the heterogeneity was high, the subgroup analysis had no significant effect on the final results. Hence, a sensitivity analysis was performed by omitting one study at a time, in order to examine the impact of each research on the estimated relative risk. The possible publication biases were identified by constructing funnel plots, in which the natural log relative risk was plotted against the SE. The meta-analysis was conducted using Cochrane Program Review Manager 5.3.

## 3. Results

### 3.1. Search Results

**Figure 2:**
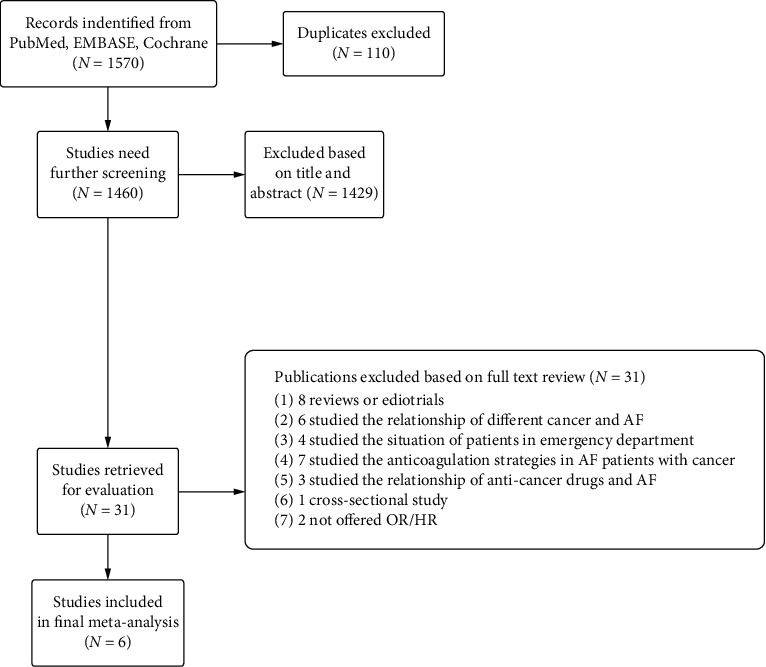
The flow diagram of the study selection process. AF, atrial fibrillation; OR, odds ratio; HR, hazard ratio.

The flow diagram for the search and selection is presented in Figure 2. Initially, a total of 1,570 records were identified using the aforementioned strategies from the PubMed, EMBASE, and Cochrane Library. Then, 110 duplicate studies were excluded. The remaining 1,460 records were qualified for further screening by title or abstract. Finally, a total of 31 potentially eligible articles were scrutinized throughout the text. Merely six articles were eventually included for the present meta-analysis.

### 3.2. Quality Assessment and Study Characteristics

Five cohort studies and one case-controlled study were included with a satisfactory NOS score. The features are presented in Table 1. The total number of participants was 533,514, and the average follow-up duration ranged within 3–19 years. Two studies only had female patients, while the other four studies had an approximately equal male/female ratio. The definition of AF and cancer was consistent in these studies.  Table 2 presents the characteristics of the patients involved in each article.

### 3.3. Meta-Analysis and Subgroup Analyses

**Figure 3:**
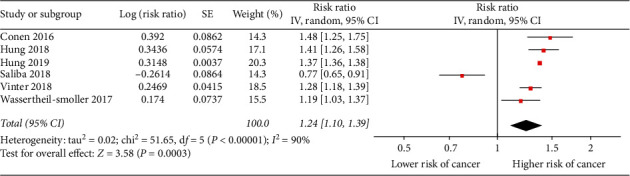
The forest plot for the combined effect quantities of the risk of cancer in AF patients. SE, standard error; IV, inverse variance.

The combined result from six separate studies revealed a link between NOAF and subsequent cancer. The summary RR was 1.24 (95% CI: 1.10–1.39, *P*=0.0003, *I*^2^ = 90%; Figure 3), indicating that patients with NOAF have an approximately 24% higher risk of cancer, when compared with non-AF patients.

Next, an analysis of the temporal trend of cancer development was performed. The RR for cancer during the initial 90 days was the highest (RR: 3.44, 95% CI: 2.29–5.57, *P*=0.00001, *I*^2^ = 88%). However, the risk declined between 90 days and one year (RR: 1.38, 95% CI: 0.90–2.12, *P*=0.14, *I*^2^ = 97%) and beyond one year (RR: 1.09, 95% CI: 0.95–1.24, *P*=0.24, *I*^2^ = 92%). Another subgroup analysis was conducted to assess the risk of three common types of cancer events, respectively. Lung cancer (RR: 1.51, 95% CI: 1.47–1.55, *P* < 0.00001, *I*^2^ = 0%) was associated with NOAF, but not colorectal cancer (RR: 1.22, 95% CI: 0.92–1.60, *P*=0.16, *I*^2^ = 92%) or breast cancer (RR: 1.10, 95% CI: 0.94–1.29, *P*=0.25, *I*^2^ = 80%). The subgroup analysis on gender revealed that both male NOAF patients (RR: 1.39, 95% CI: 1.33–1.45, *P* < 0.00001, *I*^2^ = 21%) and female NOAF patients (RR: 1.26, 95% CI: 1.11–1.44, *P*=0.00005, *I*^2^ = 78%) have a higher risk of developing cancer, when compared with non-AF patients with the same gender (Table 3).

### 3.4. Sensitivity Analysis

**Figure 4:**
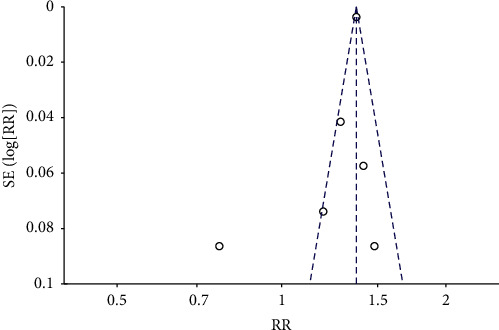
The funnel plot for all studies. SE, standard error; RR, risk ratio.

The funnel plot (Figure 4) presents the limited symmetry distribution of all the research studies, with only one research randomly beyond 95% CI, which need to be examined. That is, the study conducted by Saliba et al. [12] was the only case-control report and was influenced by potential selection bias. The integrated result was optimized (RR: 1.35, 95% CI: 1.28–1.42, *P* < 0.00001, *I*^2^ = 46%) after discarding the study conducted by Saliba et al. [12].

## 4. Discussion

Six published observational articles were incorporated into the present analysis [9–14]. The integrated result demonstrated that patients with NOAF have a 24% increased risk of developing cancer. The subgroup analysis stratified by time interval, gender, and type of cancer revealed the following: (1) the incident cancer significantly increased within 90 days after NOAF diagnosis, but not after that; (2) males appeared to have a higher risk, when compared with females; (3) the risk of lung cancer, but not colorectal cancer or breast cancer, was higher in patients with NOAF, when compared with non-AF patients.

AF is associated with increased cardiovascular morbidity and mortality, while patients with AF are exposed to a substantial risk of death due to noncardiovascular causes. The initial case-control study conducted by Muller et al. [15] reported that AF is associated with an increased occurrence of colon cancer after 5–10 years, prompting a series of studies to explore the relationship between NOAF and subsequent cancer development. However, distinct results were observed. These discrepancies may be attributable to the sample scale or selection bias in the study population. In order to systematically and comprehensively evaluate the relationship between those two entities, the present meta-analysis on NOAF and risk of cancer development was conducted for the first time.

Classic cardio-oncology focuses on the detection, monitoring, and treatment of the cardiovascular complications of chemotherapy and radiotherapy in patients with cancer. More recently, an emerging field called reverse cardio-oncology has increasingly gained the attention for patients with cardiovascular diseases who develop cancer, which significantly expands the concept of cardio-oncology [16]. The shared risk factors, oxidative stress, and inflammation signaling pathway may underlie the mutual action between cardiovascular disease and cancer [17–19]. For example, cohort studies, a meta-analysis, and a mice model study demonstrated that heart failure increased the risk of cancer development. AF and cancer share co-risk factors, such as old age, tobacco, alcoholism, obesity, diabetes mellitus, and so on [20–23]. Hung et al. [14] reported that aging, male gender, hypertension, diabetes, chronic obstructive pulmonary disease (COPD), and liver cirrhosis were significantly associated with the development of cancer among patients with AF. More intriguingly, the authors reported that there was a positive correlation between the number of risk factors and risk of cancer. The HR for cancer was 1.4 in patients with one risk factor, and this increased to 5.14 in patients with six risk factors. Furthermore, the negative effects of anxiety disorder cannot be ignored [24]. Long-term anxiety due to psychological factors or chronic diseases, such as AF, overactivates the hypothalamic-pituitary-adrenal (HPA) axis and the sympathetic-adrenal-medullary axis, elevating corticosterone and other stress hormones [25]. These hormones are not only associated with low-level systemic inflammation, leading to ultrastructural atrial remodeling [24], but also may impair immune and endocrine functions and participate in the regulation of tumor microenvironmental activity [26, 27]. In this scenario, it is applauding that AF may be a risk factor for cancer.

All six studies presented the high risk of cancer development in the first 90 days after NOAF diagnosis, while different results were observed beyond 90 days. One study revealed that an AF duration longer than 90 days is associated with reduced risk of cancer. The present meta-analysis revealed that patients with NOAF have a 24% higher risk of developing cancer. The temporal trends in the subgroup analysis demonstrated that the increased risk of cancer could be observed in the initial 90 days, while the risk declined after that. Thus, the present data did not lend support for the causal relationship between these two entities since there was no accumulative or successive impact on the cancer development in the long-term follow-up of patients with NOAF. There are several interpretations for these data. (1) AF and cancer share co-risk factors, and occult cancer might already exist before patients were diagnosed with AF. Frequent visits to the medical system for AF would increase the chance of early detection of cancer. Vinter et al. [13] reported that NOAF is closely associated with metastatic cancer within 90 days in the same year, further supporting the concept that patients with NOAF may be accompanied by occult cancers. (2) Atrial natriuretic peptide (ANP) related to AF has been shown to have extensive antiproliferative effects and might account for the significant reduction in cancer incidence after 90 days. (3) Anticoagulant therapy is the cornerstone of treatment for AF. Warfarin inhibits tyrosine kinase-dependent oncogenesis and enhances antitumor immune responses. A population-based cohort study revealed that warfarin lowers cancer incidence [28]. Thus, warfarin could counteract the oncogenesis induced by AF.

Another subgroup analysis was conducted to assess the association between cancer subtypes and AF. It was found that lung cancer is associated with NOAF but not colorectal cancer or breast cancer. A Danish cohort study [14] demonstrated that an increased risk of lung cancers and AF was found in subjects with high-risk behaviors, such as smoking, which are the common factors related to the development of AF, as well as lung cancer. Although radiation exposure to a patient with NOAF, such as chest X-ray or computed tomography, may trigger the malignant condition in the lungs, it is unlikely that X-ray exposure in routine clinical practice increases the risk of lung cancer within 90 days. It has been well recognized that patients with AF are prone to bleeding after anticoagulant drug therapy, especially gastrointestinal (GI) bleeding. GI bleeding is also correlated with potential pathological lesions, including inflammatory or diverticular disease, ulcers, vascular malformations, radiation enteropathy, and malignancies [29]. The study conducted by Clemens et al. [30] revealed that, for AF patients with dabigatran, the incidence of nongastrointestinal tumors was only 0.05%, while the incidence of gastrointestinal tumors was 0.5%. Thus, GI bleeding would advance the screening and intervention, resulting in the early diagnosis of colorectal cancer. Breast cancer is one of the most common malignant tumors in female patients. The regular administration of antiarrhythmic drugs may increase the risk of breast cancer in women with AF [10]. Studies have shown that digoxin has estrogen-like effects and significantly increases the risk of breast cancer in female AF patients [31, 32]. However, the present meta-analysis did not confirm the association between NOAF and colorectal cancer or breast cancer. Notably, the high heterogeneities were in the two subgroup analysis, in which the reliability of the association between NOAF and colorectal cancer remains to be verified.

There was a gender difference found in the present study. Male patients with NOAF had a 39% increased risk of developing cancer, whereas female patients had a 26% greater risk. In general, female patients with NOAF are associated with poor clinical outcomes. Two studies included in the present meta-analysis enrolled only women patients, which caused selective gender bias. Therefore, these results may not apply to the whole population.

These present findings may have relevance in the management of patients with NOAF. A notable increase in incident cancer within 90 days after NOAF diagnosis highlights that an appropriate strategy should be considered to screen for cancer for these patients, especially for the patients with a higher burden of risk factors, such as aging and smoking. To date, it remains unclear whether earlier diagnosis would improve the management of patients with NOAF.

## 5. Study Limitation

The present meta-analysis has several potential limitations that call for caution when interpreting the results. First, a small number of studies were included for the meta-analysis, and there was high heterogeneity among these studies. The study conducted by Saliba et al. [12] was a case-control study, which was prone to representative crowd bias. Second, eligible studies in the English language were included, while studies in non-English languages were missed. This would cause potential publication bias due to the limited number of studies. Third, NOAF and cancer share co-risk factors, which the investigators propose as the underlying mechanism for the association between NOAF and the subsequent cancer diagnosis. Risk factors, such as smoking, age, and alcohol consumption, are critical for the further analysis. Unfortunately, this information was not available.

## 6. Conclusion

The present systematic review and meta-analysis indicated that NOAF may increase the incidence of cancer. The risk of incident cancer was particularly elevated within 90 days after NOAF diagnosis, but not after that period.

## Figures and Tables

**Table 1:** The characteristics of studies included in the meta-analysis.

Study	Design	Location	Participants	Total, N	Excluded	Period of enrollment	Follow-up duration (median)	Covariates in an adjusted model	NOS score
Conen 2016 [9]	Prospective cohort study	USA	Female health professionals (>45)	34691	Prior AF/CA/CVD	1993–2013	19.1 (17.6–19.7)	Age, BMI, HTN, DM, smoke, race, and comorbidity	9

Wassertheil 2017 [10]	Prospective cohort study	USA	Postmenopausal women (50–79)	86046	NA	From 1994	15.9	Age, race, parity, age at first birth, and cancer-specific potential confounders	8

Hung 2018 [11]	Retrospective cohort study	Taipei, China	Individual from 2005	5130	＜20Y Prior AF/CA	2005–2010	3.4 ± 2	NA	8

Saliba 2018 [12]	Prospective case-control studies	USA and Israel	NA	19991	NA	From 1998	＞3Y	Age, sex, smoking, alcohol consumption, education, medication use, and comorbidity	8

Vinter 2018 [13]	Prospective cohort study	Denmark	NA	55101	Nonmelanoma skin cancer	1993–2013	19.7	Age, BMI, smoking duration, and alcohol consumption,	7

Hung 2019 [14]	Prospective cohort study	Taipei, China	NA	332555	＜20Y Prior CA	1996–2011	3.1 (0.97–6.53)	Age, sex, risk factors, and comorbidity	8

AF, atrial fibrillation; CA, cancer; CVD, cardiovascular disease; BMI, body mass index; HTN, hypertension; DM, diabetes mellitus; NA, not applicable; NOS, Newcastle–Ottawa Quality Assessment Scale.

**Table 2:** Patient characteristics of the five studies.

Study	Age (years)		Sex (M/F %)		Hypertension		Hyperlipidemia		Diabetes		Definition		Incident cases		Types of cancer
	NOAF	N-NOAF	NOAF	N-NOAF	NOAF	N-NOAF	NOAF	N-NOAF	NOAF	N-NOAF	NOAF	CA	NOAF	Sub-CA	

Conen 2016 [9]	58.0 (52.0–64.0)	53 (49–58)	Female	NA	610	8550	501	9988	64	870	ECG or med report	Pathology/cytology reports	1467	147	Colon/breast cancer
Wassertheil 2017 [10]	66.9 ± 7.1	63.2 ± 7.3	Female	NA	2011	25716	905	11612	325	3199	ECG or self-report	Medical diagnosis during the follow-up	4,376	198	Colorectal/breast cancer
Hung 2018 [11]	74 ± 13.6	NA	53.6/46.4	NA	3477	NA	864	NA	2981	NA	ICD-9	ICD-9	5130	330	Colon/breast/liver/lung cancer
Saliba 2018 [12]	63.6 ± 13.6	64.3 ± 13.6	23.6/76.4	NA	4164	4868	3196	3990	1820	2147	ECG	Pathology/cytology reports	890	352	Colorectal/breast cancer
Vinter 2018 [13]	56.2 (52.7–60.4)	NA	47.6/52.4	NA	NA	NA	NA	NA	NA	NA	ICD-8/10 or med report	ICD-10	2776	533	Prostate/lung/colorectal/breast cancer
Hung 2019 [14]	70.8 ± 13.1	NA	55.2/44.8	NA	227956	NA	83207	NA	94515	NA	ICD-9	ICD-9	332555	22911	10 kinds of specific cancers

NOAF, new-onset atrial fibrillation; N-NOAF, not new-onset atrial fibrillation; Sub-CA, subsequent cancer; NA, not applicable; ECG, electrocardiography; ICD, International Classification of Diseases; Med, medicine.

**Table 3:** The subgroup analysis of the association between AF and CA..

Study	Subgroup	Number of studies	RR	Meta-analysis		Heterogeneity *I*^2^ (%)	Test for subgroup differences *I*^2^ (%)
				95% CI	*P* value		

Gender	Male	3	1.39	1.33, 1.45	<0.00001	21	44.7
	Female	3	1.26	1.11, 1.44	0.0005	78	

Subtype of cancer	Colorectal cancer	6	1.22	0.92, 1.60	0.16	92	87.9
	Lung cancer	4	1.51	1.47, 1.55	<0.00001	0	
	Breast cancer	5	1.10	0.94, 1.29	0.25	80	

Time interval between CA diagnosis and AF	＜3M	4	3.44	2.29, 5.17	<0.00001	88	92.9
	3–12M	4	1.38	0.90, 2.12	0.14	97	
	＞12M	4	1.09	0.95, 1.24	0.24	92	

AF, atrial fibrillation; CA, cancer; RR, risk ratio.

## Data Availability

The data underlying this study are available within the article and in Supplementary Materials.
